# Multi-task learning to detect suicide ideation and mental disorders among social media users

**DOI:** 10.3389/frma.2023.1152535

**Published:** 2023-04-17

**Authors:** Prasadith Buddhitha, Diana Inkpen

**Affiliations:** School of Electrical Engineering and Computer Science, University of Ottawa, Ottawa, ON, Canada

**Keywords:** social media, suicide ideation, mental disorders, natural language processing, deep learning, multi-task learning

## Abstract

Mental disorders and suicide are considered global health problems faced by many countries worldwide. Even though advancements have been made to improve mental wellbeing through research, there is room for improvement. Using Artificial Intelligence to early detect individuals susceptible to mental illness and suicide ideation based on their social media postings is one way to start. This research investigates the effectiveness of using a shared representation to automatically extract features between the two different yet related tasks of mental illness and suicide ideation detection using data in parallel from social media platforms with different distributions. In addition to discovering the shared features between users with suicidal thoughts and users who self-declared a single mental disorder, we further investigate the impact of comorbidity on suicide ideation and use two datasets during inference to test the generalizability of the trained models and provide satisfactory evidence to validate the increased predictive accurateness of suicide risk when using data from users diagnosed with multiple mental disorders compared to a single mental disorder for the mental illness detection task. Our results also demonstrate different mental disorders' impact on suicidal risk and discover a noticeable impact when using data from users diagnosed with Post-Traumatic Stress Disorder. We use multi-task learning (MTL) with soft and hard parameter sharing to produce state-of-the-art results for detecting users with suicide ideation who require urgent attention. We further improve the predictability of the proposed model by demonstrating the effectiveness of cross-platform knowledge sharing and predefined auxiliary inputs.

## 1. Introduction

Suicide and mental illnesses are formidable challenges faced by the whole world. In 2019, 1.3% of all deaths were due to suicide, which was one of the leading causes worldwide. The suicide rate varies from country to country. In some countries, it is around two deaths per 100,000 persons, while others have reported around 80 deaths. The reported figures are for all gender and age groups. Globally, more suicides have been reported in countries with low to middle income. When considering the age groups, more than 50% of the suicides were committed by individuals before they were 50 years old (World Health Organization, 2021).

In Canada, 4,012 suicides were reported in 2019, out of which 3,058 were male. Overall, for every 100,000 persons, 10.7 suicides were reported, and according to gender, it was 16.4 males and 5.0 females. To further emphasize the severity of the suicide problem, the Canadian community health survey has reported that one in every 10 Canadians above 15 years of age has thought about suicide during their lifetime. Adversely, around 27% of the First Nations people living in reserve areas and above 15 years of age have thought about suicide in their lifetime (Statistics Canada, 2020). Given the before mentioned facts and a series of tragic events that took place at the University of Ottawa, where five students took their lives within less than a year (Dubé, 2020; Yogaretnam, 2020) highlights the relevance of early detection of suicide ideation.

When evaluating the suicide risk factors, it has been identified that mental disorders strongly correlate with suicide attempts. Even though adequate research has not been conducted to position mental disorders based on their impact on suicide attempts, Nock et al. (2009) identified that being diagnosed with a mental illness increases the risk of suicidal behavior. Extensive research has been conducted to identify the correlation between mental disorders and suicide ideation, where research on post-traumatic stress disorder (PTSD) (LeBouthillier et al., 2015), bipolar (Dome et al., 2019), depression (Hawton et al., 2013), and schizophrenia (Zaheer et al., 2020) have shown that certain mental disorders are strongly associated with increased levels of suicidality. Further research has been conducted to identify the impact comorbid conditions have on suicide and shown that comorbidity of disorders or having more than one disorder at a given time increases the risk of suicide (Holmstrand et al., 2015; Brådvik, 2018). Because 90% of the people who committed suicide were diagnosed with one or more mental disorders (Bertolote and Fleischmann, 2002) does not signify that an individual diagnosed with a mental disorder will be suicidal (Brådvik, 2018) or anyone with suicidal thoughts is diagnosed with one or more mental disorders. To identify and evaluate the before-mentioned relationship between mental disorders and suicide ideation, we conducted several experiments using three datasets representing users who self-reported diagnoses of single or multiple mental disorders and users identified with different levels of suicide risk. Even though our research aims to identify the shared feature space between users diagnosed with mental disorders and suicide ideation, the task-specific layers in the proposed multi-task learning architecture enable the discovery of features unique to the particular mental disorder or suicide ideation. Given the impact mental disorders have on suicide ideation, it could be argued that detecting users with mental illnesses is as essential as predicting suicide risk, where early detection and treatment of users with mental disorders could reduce the severe impact on one's mental and physical wellbeing and also the risk of suicide.

In general, the mental health of people worldwide is in decline (World Health Organization, 2022). For example, in Canada, 5% of a decline in mental health from 2015 to 2019 was identified among people aged 12 years and above. When analyzing the type of mental illnesses, 14% of the people were diagnosed with mood and anxiety disorders (Statistics Canada, 2020). One of the critical reasons for mental health decline is not having access to the necessary care when diagnosed with mental disorders (World Health Organization, 2022). In addition to the cost of treatment and lack of information on how to get the necessary support, social stigma and discrimination have also prohibited people from getting the required treatments and social support (World Health Organization, 2018). World Health Organization (2021) introduced four mediations to prevent suicide, out of which early identification of suicidal behavior, assessment, management, and follow-up was collectively considered as one critical point. Similarly, World Health Organization (2004) have identified early detection and treatment as critical prevention mechanisms to lower mental illnesses' impact on society.

As a preliminary step in the intricate process of preventing the adverse impact mental disorders and suicide have on society, we have focused our research on developing a model that could early detect social media users at risk of mental disorders and suicide ideation. We used user posts (especially text data) from social media platforms (Reddit and Twitter) as the primary data source, considering how these platforms have revolutionized the way people interact as a society and have become an integral part of the everyday life of many. As individuals have started sharing their day-to-day activities on these platforms, the data extracted could reveal invaluable insights into one's cognition, emotion, and behavioral aspects. Our research explores the feasibility of applying deep learning methods and, specifically, multi-task learning to predict users with suicide ideation and single or multiple mental disorders. We opted for multi-task learning, given the nature of the tasks where individuals with suicidal thoughts are more likely to be diagnosed with single or multiple mental disorders (comorbidity). The experiments were further extended to identify the effectiveness of cross-platform knowledge sharing using data from multiple social media platforms (Reddit and Twitter) with distinct distributions. Also, we explore the impact auxiliary inputs, specifically the ones discovered through exploratory analysis, have on mental illness and suicide ideation detection outcomes.

It is important to note that detecting and treating mental disorders and individuals with suicide ideation is a complex clinical process. However, considering the complexities and skills required, predicting suicide ideation and mental illnesses among individuals using natural language processing and machine learning algorithms can be considered a preliminary step in generating awareness rather than deriving conclusions on one's mental state.

The major contributions of our research are as follows:

- We use multi-task learning with both soft and hard parameter sharing to explore the bidirectional relationship between mental illnesses (either single or multiple disorders) and suicide ideation using two datasets with different distributions (without combining the datasets into a single dataset).
- We use data from two social media platforms (Twitter and Reddit) having different distributions to identify if knowledge can be successfully shared between suicide ideation and mental illness detection tasks.

## 2. Related work

Text extracted from social media platforms such as Twitter, Facebook, Reddit, and other equivalent forums has been successfully used in various natural language processing (NLP) projects to identify users with different mental disorders and suicide ideation. Social media text was used to classify users with insomnia and distress (Jamison-Powell et al., 2012; Lehrman et al., 2012), postpartum depression (De Choudhury et al., 2013b,a, 2014), depression (Schwartz et al., 2014; Resnik et al., 2015b, 2013; Tsugawa et al., 2015), Post-Traumatic Stress Disorder (PTSD) (Coppersmith et al., 2014b,a), schizophrenia (Loveys et al., 2017), and many other mental illnesses such as Attention Deficit Hyperactivity Disorder (ADHD), Generalized Anxiety Disorder, Bipolar Disorder, Eating Disorders, and obsessive-compulsive disorder (OCD) (Coppersmith et al., 2015a). The past research on mental illness detection using text data was based on features from language's lexical (e.g., character n-grams) (Malmasi et al., 2016), syntactic (e.g., part-of-speech tags) (Wang et al., 2021) and semantic (e.g., post embeddings, topic modeling) (Preotiuc-Pietro et al., 2015; Resnik et al., 2015a; Kim et al., 2016) structures. Also, features based on behavioral (Coppersmith et al., 2014b; Tsugawa et al., 2015) and emotional (De Choudhury et al., 2013b) indicators have managed to improve overall model performances. To evaluate the implications of the emotional, linguistic and cognitive facets presented in the text, many have used Linguistic Inquiry Word Count (LIWC) (Pennebaker et al., 2015). For most of the research where manually engineered features were used, the Support Vector Machine (SVM) algorithm (Cortes and Vapnik, 1995) stands out compared to other machine learning algorithms. With the advancements in neural network-based algorithms and reliable data sources, research has been conducted successfully to detect mental disorders and suicide ideation. Kshirsagar et al. (2017) have used Recurrent Neural Networks (RNNs) with attention to detect social media posts resembling crises. Husseini Orabi et al. (2018) demonstrated that using Convolution Neural Network (CNN) based architectures produces better results than recurrent neural network-based architectures when detecting social media users susceptible to depression. The effectiveness of multi-task learning with hard parameter sharing to detect suicide risk and mental health was demonstrated by Benton et al. (2017) while Buddhitha and Inkpen (2019) used a multi-channel CNN in a multi-task learning environment with predetermined auxiliary inputs to predict users with PTSD and depression.

During CLPsych 2019 (Zirikly et al., 2019) and CLPsych 2021 (MacAvaney et al., 2021) shared tasks, the participants produced results using either traditional machine learning or deep learning algorithms where logistic regression, SVM, CNN, and RNN based architectures were widely used. Manually engineered features were used to produce the best results in CLPsych 2021 with a weighted ensemble approach (Bayram and Benhiba, 2021) and a Bayesian model (Gamoran et al., 2021) while Matero et al. (2019) used RNN-based architectures and Mohammadi et al. (2019) used a fusion approach where RNN-based architectures were combined with CNN and SVM models to produce the best results at CLPsych 2019.

One of the main reasons for the continuous use of traditional machine learning methods could be the dataset size (Morales et al., 2021), where training a deep neural network with limited data could make the model overfit and not generalize well on the unseen data. The need for an interpretable outcome can also be highlighted as a valid reason for the continuous use of traditional machine learning systems in the mental illness and suicide ideation detection domain. In general, a clear distinction in the lexical and syntactic structure of the language used by individuals with different mental disorders and suicide ideation against neurotypicals can be found throughout the literature. Considering the previous and current research trends in mental illness and suicide ideation detection, we could identify that using neural network models in situations permissible could be more intuitive than using classical machine learning methods with manually engineered features.

## 3. Materials and methods

### 3.1. Ethics statement

We obtained the ethics approval certificate for the CLPsych 2015 dataset and the University of Maryland Reddit Suicidality Dataset. We provided a signed data usage agreement to acquire the Self-Reported Mental Health Diagnoses dataset from Georgetown university. During our research, we followed strict ethical guidelines to ensure the anonymity and privacy of the data. Our research does not involve any intervention and has focused mainly on the applicability of machine learning models in determining users susceptible to mental disorders and suicide ideation using the before mentioned datasets. Also, we have not included examples from the datasets in any of our publications.

### 3.2. Datasets

#### 3.2.1. The University of Maryland Reddit Suicidality dataset

The University of Maryland Reddit Suicidality dataset (hereafter known as the UMD dataset) (Shing et al., 2018) is one of the most reliable datasets assessed by clinicians and is also being used in one of the leading workshops prioritizing research in computational linguistics and clinical psychology. It is a collection of users who have published posts in the SuicideWatch subreddit. In addition to the posts published in the subreddit, the authors have collected all the posts published in Reddit by the selected users. We used a subset of the UMD dataset for our experiments, also made available through the CLPsych 2019 shared task on “Predicting the degree of suicide risk in Reddit posts” (Zirikly et al., 2019). Unlike the dataset released by Shing et al. (2018), the CLPsych 2019 dataset does not contain expert annotations but only crowdsource annotations. The dataset is annotated based on the level of suicide risk, and the risk categories are: “No risk,” “Low risk,” “Moderate risk,” and “Severe risk.” During inference (using the CLPsych 2019 shared task test dataset), the proposed models were evaluated against the CLPsych 2019 shared task best results. In addition, we evaluated our models on the data annotated by the experts to see to what extent the models trained using our proposed architecture can be generalized.

Table 1 states the details about the dataset with the number of users in each class and indicates whether it is annotated by an expert or by crowdsourcing.

**Table 1:** UMD reddit suicidality dataset.

**Annotator**	**Number of users**					
	**No risk**	**Low risk**	**Moderate risk**	**Severe risk**	**Control**	**Total**
Crowdsource	159	63	141	258	621	1,242
Expert	36	50	115	44	245	490
Total	195	113	256	302	866	1,732

Even though the inter-annotator agreement for the crowdsourced data is low, the disagreement between the expert annotations and the crowdsourced annotations is less given the flagged/not flagged and urgent/not urgent tasks. According to Table 2, which represents 245 users annotated by both crowdsourced and expert annotators, Shing et al. (2018) has identified that the disagreement between the two groups of annotators is due to misclassifying low-risk users into the higher-risk categories.

**Table 2:** Crowdsourced against expert annotations.

**Expert annotations**	**Crowdsourced annotations**			
	**No risk**	**Low risk**	**Moderate risk**	**Severe risk**
No risk	29	1	1	5
Low risk	11	13	20	6
Moderate risk	6	11	47	51
Severe risk	1	1	8	34

According to the confusion matrix (Table 2), which lists crowdsourced annotations against the expert annotations, we could identify an F1-score (for the positive class) of 0.9385 and 0.8458 for the tasks flagged/not flagged and urgent/not urgent, respectively. Considering these scores, we could identify strong inter-annotator reliability between the expert and crowdsourced annotations.

The CLPsych 2019 shared task focused on three subtasks. Our experiments are based on “task B,” where we try to predict the level of suicide risk by taking into account all the posts published in Reddit by the filtered SuicideWatch subreddit users. The task provided us with sufficient data to conduct our research. However, we did not pursue “task A” due to a lack of data, and “task C” as it focused mainly on posts published outside SuicideWatch. Even though “task B” is to predict the level of risk, our main objective is to identify the possibilities of extracting a shared feature space between the users with suicidal thoughts and mental disorders to improve the predictability of users with a mental disorder or suicide ideation. To prove our hypotheses, we selected two subtasks from “task B,” which is to distinguish users with suicide ideation from the ones that do not have suicidal thoughts (classification task of flagged/not flagged) and to distinguish the users with suicide ideation that requires urgent attention from the ones that does not (classification task of urgent/not urgent).

For the binary classification task “flagged/not flagged,” the users annotated as having “Low,” “Moderate,” and “Severe” risks' were combined to form the positive class, while the users with “No” risk were considered as the control group. Similarly, for the binary classification task of predicting urgent/not urgent users, the users belonging to the “Moderate” and “Severe” risk categories were combined to form the positive class, while the users belonging to the “No” and “Low” risk groups were combined to form the control group. To accommodate the difference between the positive and control groups, we randomly selected users from the Table 1 control group. A detailed distribution of the classes for both crowdsourced and expert annotated data is mentioned in Table 3.

**Table 3:** UMD reddit suicidality dataset with detailed class distributions.

**Class label**	**Flagged/not flagged**			**Urgent/not urgent**		
	**Train**	**Test**	**Test (expert)**	**Train**	**Test**	**Test (expert)**
No risk	127	32	36	127	32	36
Low risk	50 (+)	13 (+)	50 (+)	50	13	50
Moderate risk	113 (+)	28 (+)	115 (+)	113 (+)	28 (+)	115 (+)
Severe risk	206 (+)	52 (+)	44 (+)	206 (+)	52 (+)	44 (+)
Random control	242	- -	–	142	–	–
Total	738	125	245	638	125	245

The (+) sign indicates the positive class.

Even though we balanced the positive and the negative class distributions by randomly selecting control users, we did not balance the test dataset for a fair comparison with the state-of-the-art results published by the CLPsych 2019 shared task participants.

#### 3.2.2. Self-reported Mental Health Diagnoses dataset

We selected the Self-reported Mental Health Diagnoses dataset (hereafter known as the SMHD dataset) (Cohan et al., 2018), annotated with nine mental illnesses and a control group, to investigate the correlation between mental disorders and suicide ideation. The nine mental disorders include Autism, Attention-Deficit Hyperactivity Disorder (ADHD), Post-traumatic Stress Disorder (PTSD), Obsessive-Compulsive Disorder (OCD), Bipolar Disorder, Schizophrenia, Eating Disorder, Anxiety, and Depression. A single user has reported either one or more mental disorders, and a maximum of six were identified from specific users, which helps us discover the impact of psychiatric comorbidity on suicide ideation. The dataset contains predefined splits for training, development and testing that authors have used in their preliminary experiments. Table 4 presents the number of users under each mental disorder for each data partition.

**Table 4:** SMHD dataset with the number of users under each mental disorder and data partition.

	**Number of users**			
**Disorder**	**Train**	**Development**	**Test**	**Total**
Autism	479	480	517	1,476
ADHD	1,768	1,747	1,779	5,294
PTSD	528	516	558	1,602
OCD	409	477	390	1,276
Bipolar	1,216	1,182	1,247	3,645
Schizophrenia	238	278	267	783
Eating	104	115	112	331
Anxiety	1,711	1,593	1,675	4,979
Depression	2,662	2,574	2,611	7,847
Control	92,725	92,420	94,415	279,560
Total	101,840	101,382	103,571	306,793

**Figure 1:**
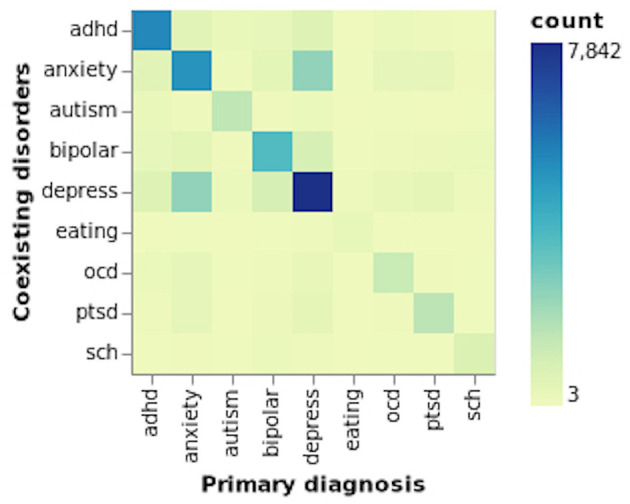
Coexisting mental disorders.

From all three partitions, it could be identified that more than 90% of the users are from the control group, followed by users susceptible to having depression, ADHD and anxiety. The minority classes include eating and schizophrenia disorders, representing 0.1 and 0.2%, respectively. The SMHD dataset consists of users who self-reported diagnoses (hereafter referred to as “diagnosed") of multiple mental disorders, where selecting a user as an input does not guarantee that a mental disorder's impact on suicide ideation is from a single disorder. The majority of such users were identified as having both “anxiety and depression” followed by “bipolar and depression” and “ADHD and depression.” For users who self-declared three mental disorders, the most common disorders to be identified were “ADHD, anxiety and depression.” Due to this reason, we conducted several experiments whereby selecting users who self-declared a single disorder or users who self-reported a primary and one or more mental disorders. From the content published by users diagnosed with multiple mental disorders, we could further establish the impact comorbidity of mental disorders have on suicide ideation (Simpson and Jamison, 1999; Nock et al., 2009; Hawton et al., 2013; Zaheer et al., 2020). Figure 1 illustrates the self-reported individual mental disorders and the coexisting disorders.

We did not use the entire dataset but only a random sample from the combined train, development and test datasets. Since we have used two different datasets (UMD and SMHD) created by two research groups for two different tasks (suicide ideation detection and mental illness detection), we had to reorganize the datasets to align the tasks (even though we do not have the same users in the two tasks) within the MTL environment. The reorganization steps of the datasets were implemented according to the proposed MTL with soft and hard parameter sharing architectural requirements and to accommodate the primary research objective, which is to identify the relationship between mental disorders and suicide ideation.

Each task in the multi-task learning architecture comprises binary classification tasks to detect whether the Reddit user has suicide ideation (flagged/not flagged and urgent/not urgent) or a mental disorder. For each of the experiments, a mental disorder was selected from the eight mental disorders (without the “eating disorder") mentioned in Table 4. We did not use users who self-declared “eating disorder” as their primary diagnosis but considered it a coexisting disorder due to insufficient instances. The selected users are matched with an equal number of control users and concatenated into a single data frame to be used as the data source when selecting random samples to train, validate and test the proposed MTL model. The number of users to select from the SMHD dataset is based on the data provided to the CLPsych 2019 shared task participants. For example, when training the proposed model to predict users with suicide ideation and who require urgent attention, we randomly selected 638 users (319 with a mental disorder and 319 for the control group) from the SMHD dataset for training and validation and 125 users for testing. Out of the 125 users, 80 self-reported a mental disorder, while 45 did not.

#### 3.2.3. Twitter mental illness detection dataset

To further determine mental disorders' impact on suicide ideation detection, we used the dataset from the Computational Linguistics and Clinical Psychology (CLPsych) 2015 shared task (Coppersmith et al., 2015b). The dataset contains Twitter users labeled as being diagnosed (self-reported diagnosis) with depression, Posttraumatic Stress Disorder (PTSD), or not having either one of the mental disorders (the control group). Table 5 presents the detailed statistics of the dataset.

**Table 5:** CLPsych 2015 shared task dataset statistics.

	**Control**	**PTSD**	**Depression**
Number of users	572	246	327
Average age	24.4	27.9	21.7
Gender (female) distribution per class	74%	67%	80%

Even though the datasets are from two different social media platforms (mental illness detection data from Twitter and suicide ideation detection data from Reddit), the objective was to investigate if knowledge can be shared among the tasks when detecting suicide ideation and mental disorders using the proposed MTL environment. The task organizers have taken the same approach as Coppersmith et al. (2014b) to collect public tweets to identify users susceptible to mental disorders. The tweets are identified using their diagnostic statements. Coppersmith et al. (2014b) have demonstrated the efficacy of the method used to collect the dataset, and many researchers have used it as a reliable dataset for predicting Twitter users susceptible to PTSD or depression. Due to the impact age and gender have on mental disorders, task organizers have predicted the age and gender of the collected users by analyzing their tweets.

### 3.3. Data pre-processing

To derive a more generalized vocabulary, we used a custom script to remove URLs, @mentions, #hashtags, RTweets, emoticons, emojis, and numbers. We removed a selected set of stopwords but kept first, second, and third-person pronouns. The first-person singular pronouns are frequently used by individuals with mental disorders such as depression (Pennebaker et al., 2007). We removed all the punctuation marks from all the datasets. The NLTK tweet tokenizer was used to tokenize the tweets, while the spaCy tokenizer was used to tokenize the Reddit data. Finally, we made the text lowercase for all the datasets, expanded the contractions, and removed extra spaces, newline characters, and tabs. After applying the abovementioned steps to all the individual tweets and Reddit posts, we removed any record that returned an empty string. From the SMHD dataset, we filtered out users with <50 tokens so that the shared feature space would be deep enough to enhance the prediction outcome of the two tasks. Similarly, we removed users with <20 tokens from the CLPsych 2015 dataset.

### 3.4. Exploratory analysis

For an overview of the datasets used to detect suicide ideation and mental illnesses and to discover appropriate auxiliary inputs that can be used to improve model performances further, several analyses were conducted using the Scattertext (Kessler, 2017) and the EMPATH (Fast et al., 2016) library. For example, we selected users who self-reported PTSD and depression to be studied during the exploratory analysis stage, considering our preliminary experiments. We selected PTSD due to the improved results that we managed to obtain and depression to understand better the comparatively poor performances it produced even though the majority of the individuals who have committed suicide were diagnosed with depression (Hawton et al., 2013). To avoid any bias during inference, we used only the training and validation partitions of the respective datasets.

When comparing the most frequently used terms, a clear distinction between the users with and without suicide ideation or mental disorders can be identified. Terms such as “anxiety,” “depression,” “suicide,” “die,” “hurt,” and “alone” can be considered more indicative of the mental state of a person susceptible to having suicidal thoughts. Also, a clear distinction can be identified between the terms used by individuals diagnosed with and without PTSD. For example, terms such as “abuse,” “abusive,” “relationship,” “therapy,” “feelings,” and “pain,” could have a strong relationship to traumatic events experienced by the individual (Wilcox et al., 2009). When it comes to the most frequently used terms by individuals who self-declared depression, it is difficult to distinguish them from those used by users in the control group. This could be due to the dataset not reflecting that many depressive characteristics.

Comparing the EMPATH categories identified by users with suicidal thoughts and users diagnosed with PTSD or depression, more similarities can be discovered among users with suicidal thoughts and PTSD. To discover the likelihood of EMPATH categories being shared among users with mental disorders (single or multiple) and suicide ideation, first, we merged the users with mental disorders and suicide ideation into the positive class. The negative class contained users from the control groups. Using the merged datasets, we generated fourteen EMPATH categories for the positive and negative classes. Table 6 demonstrates the top fourteen EMPATH categories for different combinations of sampled datasets.

**Table 6:** Filtered EMPATH categories from combined datasets.

**Suicide ideation and mental disorder**	**EMPATH categories (positive class)**	**EMPATH categories (control group)**
Suicide + depression (multiple)	Health, nervousness, sadness, medical_emergency, fear, contentment, shame, domestic_work, neglect, timidity, suffering, white_collar_job, lust, hate	Negotiate, tool, computer, musical, military, technology, toy, competing, fun, weapon, beach, ship, valuable, fight
Suicide + PTSD (multiple)	Health, medical_emergency, sadness, nervousness, fear, contentment, domestic_work, horror, torment, suicide_topw, neglect, shame, suffering, sexual	Musical, negotiate, military, technology, competing, tool, fun, computer, weapon, programming, toy, ocean, gain, beach

According to Table 6, a considerable overlap between the EMPATH categories among the different permutations of users (among users with suicidal thoughts and users diagnosed with PTSD or depression with coexisting mental disorders) can be identified. For example, categories such as “health,” “medical_emergency,” “sadness,” “nervousness,” and “fear” are commonly used among all the user combinations. Also, specific categories are prioritized differently within each group of users. For example, the category “torment” is ranked higher when combined with users diagnosed with PTSD. Similarly, the term “hate” is ranked higher when combined with users diagnosed with depression. Hypothetically it could be argued that certain mental disorders share features with users having suicidal thoughts.

To gain an understanding of the use of vocabulary among Reddit users with suicide ideation (from the UMD dataset) and Twitter users diagnosed with mental disorders (users from the CLPsych 2015 dataset diagnosed with either depression or PTSD), we used the same python libraries as before to generate and analyze the most frequently used terms and EMPATH categories. Through evaluation, it could be identified that when using the Twitter data, the filtered terms/phrases are somewhat unstructured compared to the terms/phrases used by users from the Reddit social media platform. However, we could still filter terms that could provide valuable insights into differentiating users diagnosed with a mental disorder from neurotypicals. For example, “depression” and “anxiety” are shared between users with suicidal thoughts and mental illness. The purpose of using Twitter data in our research is to discover whether knowledge can be shared between tasks that use data from multiple platforms. From the exploratory analysis, we could identify that, despite the differences in the vocabularies, users with suicide ideation (from the Reddit social media platform) share several EMPATH categories with users diagnosed with mental disorders (from the Twitter social media platform). For example, the categories “health,” “medical_emergency,” “sadness,” “fear,” and “suffering” were ranked high based on the term frequencies identified under each category.

It is important to note that looking into the most frequent terms itself will not be sufficient as conclusive evidence when classifying users as having suicidal thoughts or a mental disorder, but only as indicators that could be used as features when training machine learning models. The purpose of identifying the EMPATH categories that overlap between mental illnesses and suicide ideation is to investigate the possibilities of using the determined categories as auxiliary inputs to enhance the model performance and its generalizability, specifically by improving the level of accuracy that differentiates users with suicide ideation and mental disorders from the control group. However, we did not conduct extensive experiments using all the possible combinations because our research objective is to identify the relationship between mental disorders and suicide ideation. Furthermore, the successful use of predetermined features as auxiliary inputs to enhance the predictability of models based on deep learning architectures opens the pathway to reinvestigate the use of manually engineered features (with traditional machine learning models) as auxiliary inputs in deep learning architectures.

### 3.5. Research overview

**Figure 2:**
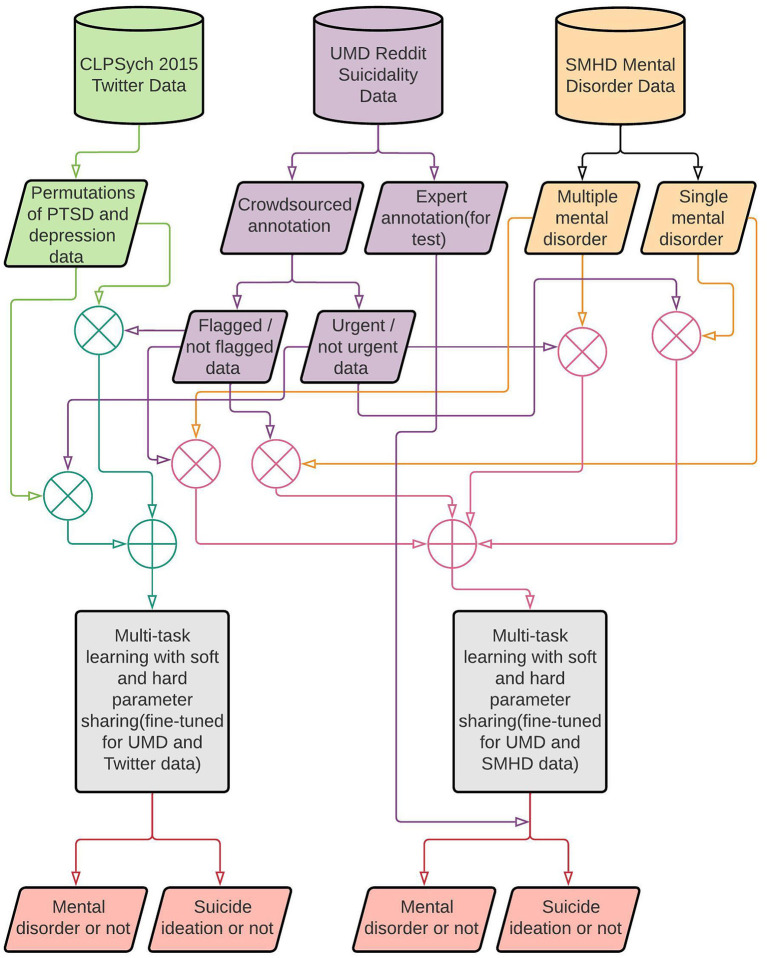
Research overview.

Figure 2 summarizes the research on mental illness and suicide ideation detection. Our research uses three datasets as mentioned above in the “Datasets” subsection (refer to Section 3.2). In the first phase of our research and as one of the tasks in the MTL environment, we used the UMD dataset divided into two parts based on the annotated risk levels. The task flagged/not flagged is implemented to identify users with and without suicide ideation, and the urgent/not urgent task is to identify users with suicide ideation that require urgent attention. We used the crowdsource annotated data to train, validate, and test our models and the expert annotated data as an additional test set to investigate further the models' generalizability (without re-training them). The SMHD dataset was used for the second task to conduct two separate experiments based on whether the user has self-declared a single or multiple mental disorder. In the second phase, we used the CLPsych 2015 dataset for the second task in the multi-task learning environment to discover the shared features between users with suicide ideation and mental disorders. The Twitter dataset consisted of different combinations of data samples with users diagnosed with PTSD or depression.

### 3.6. Model architecture

In recent years, there has been an increase in research that uses deep learning methods to detect suicide ideation and mental disorders using publicly available social media data. However, to the best of our knowledge, not much research has used multi-task learning models to detect mental illness or suicide ideation. Also, we have not identified any research on detecting suicide ideation and mental disorders using multi-task learning with two different datasets submitted in parallel to a single model. Given the challenges faced when creating a dataset to predict mental disorders (Coppersmith et al., 2014b) or suicide ideation (Shing et al., 2018), it will be complicated to construct a dataset with the same users with mental disorders and suicide ideation. Also, having fewer datasets with limited data points makes using multiple datasets in parallel with MTL a more viable solution.

Unlike related literature, where datasets were combined to form a single dataset with users having mental disorders or having attempted suicide (Benton et al., 2017), we submitted the datasets independently to our proposed architecture to predict users with mental disorders or suicide ideation. Hypothetically, combining users with mental disorders and suicide ideation (if the initial annotations were only for a single task and the combined datasets were not re-annotated accordingly) could introduce invalid narratives where it has been identified that users with suicide ideation could be diagnosed with different mental disorders such as PTSD (Wilcox et al., 2009; LeBouthillier et al., 2015), bipolar disorder (Simpson and Jamison, 1999; Dome et al., 2019) and mood disorders such as depression (Bertolote and Fleischmann, 2002; Bertolote et al., 2004). Also, when combining datasets containing users with mental disorders and suicide ideation, certain users within the suicide dataset could have been diagnosed with either one or more mental disorders. Such circumstances could allow the model to penalize features strongly associated with suicide ideation and mental disorders.

**Figure 3:**
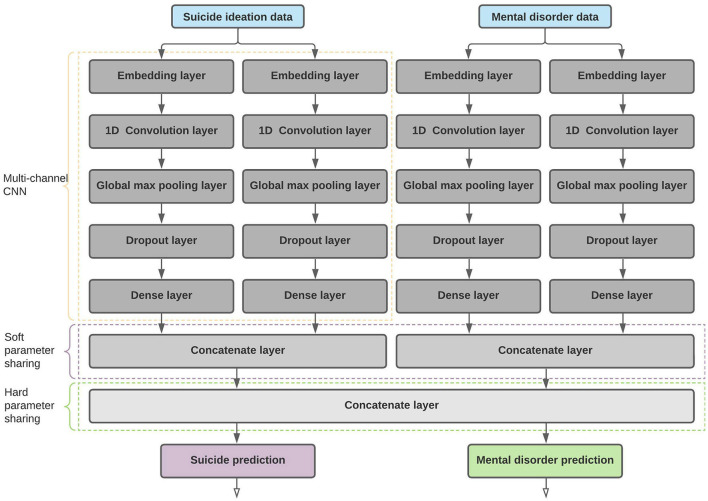
Proposed MTL architecture with soft and hard parameter sharing.

We used multi-task learning because it aims to generalize the primary task by sharing representations of related tasks (Caruana, 1997). MTL approaches can be categorized mainly into two types based on how the parameters are shared. One approach is hard parameter sharing, where model weights are shared between the tasks (Caruana, 1997). The second approach is soft parameter sharing, where each task is trained using its subnetwork without sharing any parameters between the layers. However, the parameters are regularized (by using a custom loss function) between the layers of the sub-models to obtain similarity (Ruder, 2017). We conducted several experiments using the before-mentioned architectures and identified that a model trained using a combination of soft and hard parameter sharing produced better results on unseen data. The architecture of the best-performing model is mentioned in Figure 3.

After selecting the most fitting MTL architecture, we performed suicide ideation and mental illness detection using several combinations of datasets. The combinations are based on self-reported mental disorders where certain users are diagnosed with either single or multiple mental illnesses. We divided the experiments into two streams where one is to predict users with suicide ideation or a mental disorder using the posts from users diagnosed with a single mental disorder, and the second is to use the posts from users with multiple mental disorders.

We used a multi-channel Convolutional Neural Network (Kim, 2014) as the core computational unit in our proposed architecture. Throughout related literature and especially in research on mental health using social media data, we identified that many researchers have trained models based on the CNN architecture. For example, Husseini Orabi et al. (2018) and Buddhitha and Inkpen (2019) have demonstrated the effectiveness of using CNN over RNN-based architectures when predicting users for mental disorders, and Cohan et al. (2018) and Shing et al. (2018) used a CNN architecture for their preliminary experiments. Given the limited training data, we used only two channels to reduce model complexity, which will reduce model overfitting. We also discovered that a CNN architecture with a single channel underfit the training data and performed poorly on the holdout datasets. For each CNN channel processing either suicide ideation or mental disorder data, we used two different kernel sizes to get posts processed with different n-gram (sequence of words) sizes. Each CNN channel was initialized with kernel sizes 3 and 4 (each CNN layer submitted with either suicide or mental illness data). In search of optimal kernel size, we experimented with several values, in which the kernel sizes 3 and 4 produced better results than sizes 1 and 2, 2 and 3, and 4 and 5. With the selected kernel size, each one-dimensional convolution layer contained 256 filters.

To prepare the input for the CNN layer, we used a randomly initialized and trainable embedding layer with an output dimension of 300 units. Using different dimensions for dense embeddings, such as 100, 200 and 300, we identified 300 units as the optimal embedding dimension that could enhance model predictability. In addition, we conducted several experiments with different pre-trained word embedding architectures such as fastText (Grave et al., 2018), GloVe (Pennington et al., 2014), Byte-Pair embeddings (Heinzerling and Strube, 2018), Character Embeddings (Lample et al., 2016) and stacked embeddings (Akbik et al., 2018). We determined that randomly-initialized embeddings produced better results for our tasks than the pre-trained embedding architectures, even when pre-trained weights were fine-tuned during model training. One reason for the pre-trained embeddings to perform poorly could be that the text used to train such embeddings are not closely related to the posts published by users identified with suicide ideation or mental disorders. We did not train any embedding architecture with our data, as our main objective is to discover the correlation between suicide ideation and mental disorders.

The input dimension or the vocabulary size differed based on the input text, where the vocabulary was created using the training data from both SMHD and UMD datasets. Creating the vocabulary from both datasets allows the tasks to converge into a shared feature space that could improve the overall model performances.

The output from the convolution layer is then sent through a Global Maximum Pooling layer to reduce the number of learnable parameters, and as a result, model overfitting can be minimized. Furthermore, the computational overload can also be reduced. We took several measures to regularize the network so that the impact of model overfitting, especially when using a limited number of data points, can be reduced. For example, we used dropout (Srivastava et al., 2014), where the output from the CNN layers will be randomly assigned with zeros and L1 and L2 regularization to penalize layers for having larger weights. The regularized output from the CNN layer was sent through a fully connected layer with 512 hidden units. The dense representation from each channel (two channels for each task) merged to form a task-specific representational vector. The merged vectors are used during soft parameter sharing, where the distance between parameter vectors is regularized using mean squared error to discover the similarity between tasks.

Each task-specific representational vector is concatenated to form the shared representation layer containing 2,048 features. The shared representation is used as an input to two softmax layers to generate the class probabilities for each task. Because the parameters are optimized on multiple tasks (during hard parameter sharing) instead of a single task, the negative impact of overfitting can be further reduced.

#### 3.6.1. Proposed architecture with auxiliary inputs

To identify whether or not auxiliary inputs can be used to enhance the model performances, we extended the proposed architecture mentioned in Figure 3 to be tested with multiple inputs discovered during the exploratory analysis (refer to Section 3.4).

In addition to the text inputs from the users with suicide ideation and mental disorders, the EMPATH categories extracted from the content posted by users with suicide ideation and mental disorders are used to identify the impact of such auxiliary inputs on prediction outcomes. After scaling the extracted EMPATH features, we transformed the given auxiliary input by sending it through a densely connected layer. The number of hidden units used with the dense layer differed based on the task. When predicting users with and without suicide ideation and mental disorder, we used eight hidden units, and the model performances decreased with additional units. To detect users with suicide ideation who require urgent attention, we identified 16 units as the optimal number of hidden units. However, when conducting the experiments to prove the domain adaptation capabilities of the proposed model where Reddit posts were used to detect suicide ideation and tweets to detect users with mental disorders, we used a dense layer with 32 hidden units to transform the auxiliary inputs. The transformed data is merged with the multi-channel CNN outputs generated using suicide ideation and mental disorder data. Similar to the proposed architecture, the soft parameter sharing will be based on the vectors created using the previously merged outputs. Given the tasks of detecting users with suicide ideation or mental disorders, the vectors to be regularized will have 1,032 parameters. Finally, the merged vectors will be concatenated to form the hard parameter sharing layer.

#### 3.6.2. Baseline architecture

We used a multi-channel CNN with a similar configuration as the proposed MTL architecture but for single-task learning as our baseline. A similar number of hyperparameters used with the proposed architecture is used, which contains a randomly initialized embedding vector of 300 dimensions connected to a one-dimensional convolution layer with 256 filters. The multi-channel CNN network used kernel sizes 3 and 4 with global maximum pooling and dropout for network regularization. The outputs from each channel are concatenated and submitted to a softmax layer to generate the class probabilities.

#### 3.6.3. Cross-platform knowledge transfer

Transfer learning is one of the extensively proven deep learning approaches to sharing knowledge between similar tasks. With transfer learning, one or more pre-trained layers on a similar task can be used with the task of interest so that certain low-level features can be shared between the two tasks (Goodfellow et al., 2016). Even with the proven success of applying transfer learning methods, it has yet to produce reliable results when using data annotated with proxy-based methods (Harrigian et al., 2020). In addition to using data from the same platform, we identify the adaptability of cross-platform knowledge sharing using multi-task learning instead of transfer learning. The previously proposed architectures based on multi-task and single-task learning will be used to prove such architectures' effectiveness when discovering shared knowledge between different social media platforms. Due to the limited data points provided with the CLPsych 2015 Twitter dataset, we could not test individual mental disorders' impact on suicide ideation detection. However, only the impact of a collection of mental disorders (PTSD and depression) on suicide ideation detection, and also the impact suicide ideation detection has on the same mental disorders, were measured.

### 3.7. Experiments

During our research, we investigate the impact different mental illnesses have on suicide ideation and whether users diagnosed with multiple mental disorders share more features with suicide ideation than those diagnosed with a single mental disorder. All the data used in predicting suicide ideation is the same as the CLPsych 2019 shared task data, and to demonstrate the generalizability of our proposed model further, we used the data annotated by the experts, which is a part of the UMD dataset but not made available to the CLPsych 2019 shared task participants. In addition, we conduct several experiments to demonstrate the impact auxiliary inputs have on suicide ideation and mental disorder detection. We used similar experiment structures for both the research streams, flagged/not flagged and urgent/not urgent, where one is to detect users with and without suicide ideation (flagged/not flagged), and the second is to identify users with suicide ideation who require urgent attention (urgent/not urgent).

#### 3.7.1. Task: Flagged/not flagged

As mentioned in Section 3.2.1 and Table 3, we merged users in different risk categories according to the given task to generate the positive and the control user groups. Each class consisted of 369 users. As mentioned in Section 3.2.2, we randomly sampled 369 users from each mental disorder (from eight mental disorders) to create the positive class for mental illness detection. As for the control group, a matching number of users from the provided control dataset were randomly selected. We use the test data made available for each task to evaluate the trained model. For suicide ideation detection, we use 125 users (32 users for the control class and 93 users for the positive class) provided by the CLPsych 2019 shared task organizers. In addition, we select a random test set for mental illness detection that is equal to the number of samples made available for suicide ideation detection (125 users).

Even though we obtained the best results using randomly initialized embeddings, we conducted several experiments using pre-trained fastText word embeddings (with 300 dimensions) to measure the impact pre-trained embeddings have on model performances. Also, we conducted several experiments to identify the impact EMPATH categories discovered using the exploratory analysis have on model performances when used as an auxiliary input. We identified that the top 14 EMPATH categories (based on the most frequently used terms) discovered during exploratory analysis enhanced the model performances. The auxiliary inputs were used only with the best-performing model, and the categories used are as follows:“*health,” “medical_emergency,” “sadness,” “nervousness,” “fear,” “contentment,” “domestic_work,” “horror,” “torment,” “suicide_topw,” “neglect,” “shame,” “suffering,” “sexual."*

For the cross-platform experiments performed using the UMD and CLPsych 2015 Twitter datasets, we randomly selected multiple stratified samples from the CLPsych 2015 dataset to predict mental disorders. The number of instances and the class distributions are the same as in the UMD dataset. The class distributions of the sampled datasets were proportional to the original CLPsych 2015 dataset class distribution, and the following experiments were conducted using different permutations. Similar to the previous experiments, we used fastText embeddings with 300 dimensions to identify the impact of pre-trained embeddings on the model performance. In addition, we merged ten pre-determined EMPATH categories: “health,” “medical_emergency,” “crime,” “horror,” “war,” “sadness,” “fear,” “suffering,” “aggression,” “neglect” to discover the enhancements such auxiliary inputs could bring to model performances.

- suicide + more_ptsd: Proportionally using more data from users diagnosed with PTSD.
- suicide + more_depress: Proportionally using more data from users diagnosed with depression.
- suicide + ptsd_depress: Proportionally using data from users diagnosed with depression and PTSD.
- suicide + ptsd_depress + embed: Proportionally using data from users diagnosed with depression and PTSD combined with pre-trained word embeddings.
- suicide + ptsd_depress + empath: Proportionally using data from users diagnosed with depression and PTSD combined with EMPATH categories.
- baseline (ptsd + depress): Proportionally using data from users diagnosed with depression and PTSD to compute the baseline for the mental illness detection task.

#### 3.7.2. Task: Urgent/not urgent

A similar procedure as before was followed when selecting data to predict users with suicide ideation that requires urgent attention. The only difference between the two tasks is the sample size, where users from both “No” risk and “Low” risk categories are merged into the control group. We selected 319 users from the “Moderate” and “Severe” risk categories to create the positive class. The negative class contains users from the “No” risk and “Low” risk categories that add up to 177 users. We randomly selected 142 users from the UMD control group to upsample the negative class. Similar to the flagged/not flagged task, we selected two user groups with single or multiple mental disorders. The test dataset for suicide ideation detection was kept untouched, where it contained 125 users, 45 users in the control group and 80 users labeled as positive. To match the UMD test dataset, we randomly selected data from the SMHD dataset during inference (making predictions on unseen test data using the trained model).

Like the flagged/not flagged task, we conducted several experiments using the pre-trained fastText word embeddings. To identify the impact EMPATH categories have on predicting users with suicide ideation or a mental disorder, we experimented with several categories from the top 14 discovered during exploratory analysis. With limited experiments, the categories mentioned below produced the best results out of the limited experiments conducted using EMAPTH categories as auxiliary inputs. Even though we could not identify any improvement over the results when not using such categories, more opportunities exist to conduct extensive experiments using different combinations of the categories.

“*neglect,” “anger,” “sadness,” “torment,” “emotional,” “shame."*

Similar to the task flagged/not flagged, we randomly selected stratified samples from the CLPsych 2015 dataset following the same class distribution of the UMD dataset. The subsequent experiments were completed using combinations of data representing different mental disorders (PTSD and depression) to identify mental disorders' impact on suicide ideation. In addition to using the fastText word embeddings with 300 dimensions, the same EMPATH categories used with the task flagged/not flagged were used in some experiments.

- suicide + more_ptsd + embed: Proportionally using more data from users diagnosed with PTSD and the remaining from those diagnosed with depression. Use pre-trained word embeddings.
- suicide + more_ptsd + empath: Proportionally using more data from users diagnosed with PTSD and the remaining from those diagnosed with depression. Use EMPATH categories as auxiliary inputs.
- baseline (more_ptsd + depress): Proportionally using more data from users diagnosed with PTSD and the remaining from those diagnosed with depression to compute the baseline for the mental disorder detection task.

#### 3.7.3. Baseline

To calculate the baseline, we separated each task of the MTL environment and trained different models using a multi-channel CNN network as mentioned in Section 3.6.2. For suicide ideation detection, we used the same dataset used in the MTL environment for the two tasks (flagged/not flagged and urgent/not urgent). In addition to using the UMD crowdsourced and expert annotated data during inference, we calculated a baseline using the expert annotated data to approximate how well our model trained on crowdsourced data has generalized on the expert annotated data. To calculate a baseline for the users who self-declared mental disorders (using the SMHD dataset), we experimented: for each task (for the tasks of flagged/not flagged and urgent/not urgent), for each mental disorder (for eight mental disorders) and also for multiple or single mental disorder diagnosis. For the experiments that use the CLPsych 2015 dataset and for the task flagged/not flagged, we used a stratified sample of users diagnosed with PTSD and depression. For the task urgent/not urgent, we selected more instances from the PTSD class for training, considering the better results determined during preliminary experiments.

#### 3.7.4. Model training

The data frame containing the pre-processed and normalized (to have a uniform sequence length) data was split into five stratified shuffle splits (Pedregosa et al., 2011) with 80% of data for training and the remaining 20% for validation. Given the data split, task alignment is vital when submitting the input data to the model. For example, when fitting the model with the input data, both the datasets' positive and negative classes must be aligned so that a user with suicide ideation is in parallel with a user diagnosed with single or multiple mental disorders.

Once the input data is submitted accordingly, soft parameter sharing will be on the parameter vectors generated using data points belonging to users with suicide ideation and mental disorders. If the tasks are misaligned, the comparison will be based on a mix of positive and negative classes, increasing the loss. During the hard parameter sharing stage, the two tasks will share features from the concatenated vectors generated by users with/without suicide ideation and with/without single or multiple mental disorders. Similarly, the shared feature space would be less effective when predicting the outcome for each task if not aligned accordingly. When training the model, it was identified that the most stable learning rate is 0.001 with Adam optimizer (Kingma and Ba, 2015). The Rectified Linear Unit (ReLU) (Nair and Hinton, 2010) was applied to the output generated by both the CNN layers and the Dense layers that follow. However, when using the CLPsych 2015 dataset, we discovered LeakyReLU (Maas et al., 2013) to be more reliable when used as the activation function with an alpha (α) value of 0.2 on the outputs generated by the convolution and dense layers. To reduce model overfitting, we used dropout (Srivastava et al., 2014) with a probability of 0.5 and *L*1 and *L*2 regularization with a regularization factor (10^−5^) to penalize convolution and fully connected layers for having larger weights. When fitting the data to the proposed architecture, we ensured not to shuffle data to maintain task alignment. We used a custom loss function, which summed categorical cross-entropy loss and mean squared error. The mean squared error was used to regularize the parameter vectors of the two tasks (suicide ideation detection and mental illness detection). When training both flagged/not flagged and urgent/not urgent tasks, we experimented with several mini-batch sizes and identified that smaller batch sizes produce better results than larger batch sizes (Masters and Luschi, 2018). A batch of sizes: 8, 16 or 32 was used in the experiments, and a mini-batch of size 8 substantially improved the model performances on validation data and the trained model generalized well on the imbalanced unseen data. We trained the model for 10 epochs^1^ with early stopping if the validation loss did not improve for three epochs. In addition, we reduced the learning rate by a factor of 0.1 if the validation loss did not improve for two consecutive epochs. The minimum learning rate was initialized to be 10^−8^. The model with the lowest validation loss was returned for inference.

#### 3.7.5. Inference

The test dataset made available by the CLPsych 2019 shared task was used for inference for both the flagged/not flagged and urgent/not urgent tasks. Unlike the training dataset, the class distribution of the test dataset is imbalanced. The class distribution for the flagged/not flagged task is ~ 74% for the positive class and 26% for the negative, and for the urgent/not urgent task, it is around 64% for the positive class and 36% for the negative. We calculated macro Precision, Recall and F1 score during model evaluation. In addition, we calculated the macro averaged ROC AUC score and the accuracy to better understand the trained model's performances. Comparing the test results obtained using different models trained on stratified splits, we identified a certain level of variance which could happen due to the stochastic nature of the algorithms. Several other factors could have also contributed to the variance of the results. One key factor could be the statistical noise in the dataset (Brownlee, 2018), especially when the data is automatically annotated. To overcome the variance in the results, we used the model averaging ensemble approach (Brownlee, 2018). The ensemble approach allows the prediction outcome to be generalized, where different models trained on the same data might not make the same errors on the test data (Goodfellow et al., 2016). We used the expert annotated UMD dataset to demonstrate how well the model generalizes on unseen data. Even though a certain level of class imbalance can be identified from the expert annotated data, especially from the flagged/not flagged task, the prediction results on the test dataset annotated by the experts show that the trained model is well generalized. However, we did not use the expert annotated data to test the model trained on the CLPsych 2015 dataset due to the limited number of instances. To match the imbalanced CLPsych 2019 shared task test dataset, we randomly selected an equal number of instances from the Twitter data to be used as test data.

## 4. Results

We calculated the baseline for each task and mentioned them alongside the multi-task learning results for comparison. The baselines were calculated for each of the experiment categories (flagged/not flagged and urgent/not urgent), for each disorder (eight disorders) and for each type of disorder whether the user is diagnosed with a single mental disorder or more than one disorder in addition to their primary diagnosis. Along with the mental disorder identified with the best macro F1 score, we have mentioned the results from the experiments using the pre-trained embeddings and the auxiliary inputs. We did not conduct the experiment using the pre-trained embeddings or the auxiliary inputs with all the mental disorders but only with the disorder that produced the best performances based on the F1 score. The multi-tasks with the best results are highlighted, and the best F1 score is stated in bold font.

When using the CLPsych 2015 dataset, we selected a different number of instances from each mental disorder (from either PTSD or depression) to identify the impact each has on suicide ideation and mental illness detection. In addition to identifying the platform independence when it comes to knowledge sharing, the experiments further established the impact certain mental disorders, such as PTSD and depression, have on suicide ideation detection. We used pre-trained embeddings and EMPATH categories with the best-performing model to see if the trained model could be further generalized. We utilized the same stratified sample used with multi-task learning that produced the best F1 score to compute a strong baseline. We highlighted the best results and emphasized the best F1 score.

The results tables mentioned in the following sections consist of the columns: Multi-task (the MTL experiment), Ps (precision for suicide ideation detection), Pm (precision for mental illness detection), Rs (recall for suicide ideation detection), Rm (recall for mental illness detection), F1s (F1 score for suicide ideation detection), F1m (F1 score for mental illness detection), ACCs (accuracy for suicide ideation detection), ACCm (accuracy for mental illness detection), AUCs (area under the ROC curve for suicide ideation detection), AUCm (area under the ROC curve for mental illness detection).

### 4.1. Task: Flagged/not flagged

Table 7 demonstrates the results obtained for the following experiments: “flagged/not flagged + single mental disorder": flagged/not flagged task with users who self-declared a single mental disorder. Each row represents the results obtained for the two tasks within the multi-task learning environment, followed by the respective baselines. The last row of the section states the suicide ideation detection baseline.

**Table 7:** Experiments: flagged/not flagged with users diagnosed with a single mental disorder (flagged/not flagged + single mental disorder), flagged/not flagged with users diagnosed with multiple mental disorders (flagged/not flagged + multiple mental disorders), flagged/not flagged with multiple mental disorders and evaluated on the expert annotated data, flagged/not flagged with users diagnosed with PTSD or depression (flagged/not flagged using tweets).

**Experiments**	**Multi-task**	**Ps**	**Pm**	**Rs**	**Rm**	**F1s**	**F1m**	**ACCs (%)**	**ACCm (%)**	**AUCs**	**AUCm**
Flagged/not flagged + single mental disorder	Suicide + adhd	0.737	0.726	0.722	0.726	0.728	0.726	80.00	79.20	0.814	0.814
	Adhd baseline	–	0.686	–	0.730	–	0.694	–	73.60	–	0.837
	Suicide + anxiety	0.780	0.780	0.774	0.774	0.777	0.777	83.20	83.20	0.860	0.860
	Anxiety baseline	–	0.776	–	0.845	–	0.789	–	81.60	–	0.902
	Suicide + autism	0.742	0.742	0.691	0.691	0.708	0.708	80.00	80.00	0.829	0.827
	Autism baseline	–	0.732	–	0.757	–	0.742	–	79.20	–	0.831
	Suicide + bipolar	0.778	0.778	0.805	0.805	0.789	0.789	83.20	83.20	0.889	0.889
	Bipolar baseline	–	0.799	–	0.846	–	0.816	–	84.80	–	0.920
	Suicide + depress	0.795	0.795	0.769	0.769	0.780	0.780	84.00	84.00	0.880	0.880
	Depress baseline	–	0.730	–	0.792	–	0.738	–	76.80	–	0.876
	Suicide + ocd	0.790	0.785	0.743	0.753	0.761	0.767	83.20	83.20	0.872	0.871
	Ocd baseline	–	0.781	–	0.825	–	0.796	–	83.20	–	0.895
	Suicide + ptsd	0.831	0.831	0.831	0.831	**0.831**	**0.831**	87.20	87.20	0.944	0.946
	Ptsd baseline	–	0.828	–	0.893	–	0.848	–	87.20	–	0.946
	Suicide + sch	0.820	0.829	0.826	0.842	0.823	0.835	86.40	87.20	0.874	0.873
	Schizophrenia baseline	–	0.776	–	0.845	–	0.789	–	81.60	–	0.885
	Suicide baseline	0.647	–	0.673	–	0.654	–	71.20	–	0.726	-
Flagged/not flagged + multiple mental disorders	Suicide + adhd	0.749	0.749	0.768	0.768	0.757	0.757	80.80	80.80	0.874	0.875
	Adhd baseline	–	0.783	–	0.835	–	0.800	–	83.20	–	0.916
	Suicide + anxiety	0.785	0.785	0.753	0.753	0.767	0.767	83.20	83.20	0.892	0.893
	Anxiety baseline	–	0.799	–	0.846	–	0.816	–	84.80	–	0.930
	Suicide + autism	0.778	0.778	0.784	0.784	0.781	0.781	83.20	83.20	0.876	0.877
	Autism baseline	–	0.764	–	0.834	–	0.774	–	80.00	–	0.882
	Suicide + bipolar	0.872	0.864	0.848	0.832	0.859	0.846	89.60	88.80	0.940	0.939
	Bipolar baseline	–	0.801	–	0.856	–	0.819	–	84.80	–	0.925
	Suicide + depress	0.758	0.748	0.753	0.758	0.755	0.752	81.60	80.80	0.870	0.871
	Depress baseline	–	0.789	–	0.856	–	0.805	–	83.20	–	0.904
	Suicide + ocd	0.768	0.778	0.789	0.794	0.777	0.785	82.40	83.20	0.888	0.889
	Ocd baseline	–	0.765	–	0.814	–	0.780	–	81.60	–	0.906
	Suicide + ptsd	0.896	0.896	0.843	0.843	0.865	0.865	90.40	90.40	0.967	0.966
	Suicide + ptsd + EMPATH	0.915	0.915	0.848	0.848	**0.875**	**0.875**	91.20	91.20	0.952	0.951
	Suicide + ptsd + fastText	0.872	0.872	0.848	0.848	0.859	0.859	89.60	89.60	0.958	0.959
	Ptsd baseline	–	0.836	–	0.868	–	0.849	–	88.00	–	0.942
	Suicide + sch	0.778	0.778	0.784	0.784	0.781	0.781	83.20	83.20	0.895	0.889
	Schizophrenia baseline	–	0.743	–	0.818	–	0.739	–	76.00	–	0.922
Expert	Suicide + ptsd	0.843	0.843	0.851	0.851	**0.847**	**0.847**	92.245	92.245	0.955	0.955
	Suicide baseline (expert)	0.587	–	0.652	–	0.585	–	70.204	–	0.729	-
Flagged/not flagged using tweets	suicide + more_ptsd	0.857	0.857	0.842	0.842	0.849	0.849	88.80	88.80	0.956	0.955
	Suicide + more_depress	0.793	0.783	0.851	0.835	0.810	0.799	84.00	83.20	0.928	0.928
	Suicide + ptsd_depress	0.861	0.861	0.868	0.868	0.864	0.864	89.60	89.60	0.960	0.960
	Suicide + ptsd_depress + embed	0.846	0.836	0.873	0.868	0.858	0.849	88.80	88.00	0.946	0.946
	Suicide + ptsd_depress + empath	0.869	0.869	0.884	0.884	**0.876**	**0.876**	90.40	90.40	0.960	0.959
	Baseline (ptsd + depress)	–	0.806	–	0.877	–	0.824	–	84.80	–	0.951

The bold values represent the highest F1 score for suicide ideation and mental disorder detection.

“flagged/not flagged + multiple mental disorders": flagged/not flagged task with users who self-declared multiple mental disorders. After identifying the primary diagnosis that produced the best results, we trained separate models using the same data with pre-trained word embeddings and auxiliary inputs (EMPATH categories identified through exploratory analysis).

“expert”: flagged/not flagged with users whom self-declared PTSD and one or more other mental disorders and evaluated on the UMD expert annotated data.

“flagged/not flagged using tweets": results obtained for each of the experiments conducted to predict users with suicide ideation and mental disorders (the generic category of having a mental illness or not). The “baseline (ptsd + depress)” identifies whether a user has a mental disorder, which could be either PTSD or depression.

### 4.2. Task: Urgent/not urgent

Table 8 demonstrates the results obtained for the following experiments: “urgent/not urgent + single mental disorder": urgent/not urgent task with users who self-declared a single mental disorder. Similar to Table 7 and section “flagged/not flagged + single mental disorder,” we randomly selected users from the SMHD dataset diagnosed with a single mental disorder for the mental illness detection task. Because the best performances were reported using users diagnosed with a single mental disorder (PTSD), we used pre-trained fastText embeddings and EMPATH categories as auxiliary inputs to train two additional models to identify if such additions to the proposed architecture could enhance the overall performances of the model. Finally, the last row represents the suicide ideation detection baseline predicted using the multi-channel CNN model. “urgent/not urgent + multiple mental disorders": urgent/not urgent task with users who self-declared multiple mental disorders. “expert": urgent/not urgent task with users who self-declared PTSD only and evaluated on the expert annotated data. “urgent/not urgent using tweets": lists the experiments conducted to identify if a user has suicidal thoughts (requiring urgent attention) or mental illness using the UMD and CLPsych 2015 datasets. The “baseline (more_ptsd + depress)” identifies users with mental disorders, such as PTSD or depression.

**Table 8:** Experiments: urgent/not urgent with users self-diagnoses with a single mental disorder (urgent/not urgent + single mental disorder), urgent/not urgent with users self-diagnoses with multiple mental disorders (urgent/not urgent + multiple mental disorders), urgent/not urgent with users diagnosed only with PTSD and evaluated on the expert annotated data (expert), urgent/not urgent with users diagnosed with PTSD or depression (urgent/not urgent using tweets).

**Experiments**	**Multi-task**	**Ps**	**Pm**	**Rs**	**Rm**	**F1s**	**F1m**	**ACCs (%)**	**ACCm (%)**	**AUCs**	**AUCm**
Urgent/not urgent + single mental disorder	Suicide + adhd	0.704	0.685	0.718	0.695	0.707	0.688	72.00	70.40	0.777	0.780
	Adhd baseline	–	0.752	–	0.773	–	0.753	–	76.00	–	0.841
	Suicide + anxiety	0.785	0.785	0.750	0.750	0.761	0.761	79.20	79.20	0.842	0.843
	Anxiety baseline	–	0.800	–	0.812	–	0.804	–	81.60	–	0.876
	Suicide + autism	0.717	0.717	0.686	0.686	0.694	0.694	73.60	73.60	0.780	0.778
	Autism baseline	–	0.723	–	0.742	–	0.715	–	72.00	–	0.815
	Suicide + bipolar	0.789	0.778	0.765	0.759	0.774	0.766	80.00	79.20	0.883	0.881
	Bipolar baseline	–	0.802	–	0.822	–	0.807	–	81.60	–	0.886
	Suicide + depress	0.818	0.805	0.773	0.767	0.787	0.779	81.60	80.80	0.868	0.867
	Depress baseline	–	0.792	–	0.786	–	0.789	–	80.80	–	0.895
	Suicide + ocd	0.722	0.722	0.712	0.712	0.716	0.716	74.40	74.40	0.855	0.853
	Ocd baseline	–	0.766	–	0.788	–	0.756	–	76.00	–	0.863
	Suicide + ptsd	0.859	0.859	0.865	0.865	**0.862**	**0.862**	87.20	87.20	0.946	0.946
	Suicide + ptsd + EMPATH	0.863	0.849	0.856	0.834	0.859	0.840	87.20	85.60	0.943	0.943
	Suicide + ptsd + fastText	0.860	0.860	0.840	0.840	0.848	0.848	86.40	86.40	0.942	0.941
	Ptsd baseline	–	0.839	–	0.866	–	0.843	–	84.80	–	0.932
	Suicide + sch	0.748	0.739	0.745	0.739	0.747	0.739	76.80	76.00	0.870	0.868
	Schizophrenia baseline	–	0.808	–	0.813	–	0.810	–	82.40	–	0.897
	Suicide baseline	0.616	–	0.625	–	0.616	–	63.20	–	0.680	-
Urgent/not urgent + multiple mental disorders	Suicide + adhd	0.777	0.777	0.738	0.738	0.750	0.750	78.40	78.40	0.869	0.869
	Adhd baseline	–	0.775	–	0.769	–	0.772	–	79.20	–	0.855
	Suicide + anxiety	0.846	0.843	0.838	0.843	0.842	0.843	85.60	85.60	0.921	0.921
	Anxiety baseline	–	0.841	–	0.858	–	0.847	–	85.60	–	0.912
	Suicide + autism	0.839	0.839	0.806	0.806	0.818	0.818	84.00	84.00	0.901	0.900
	Autism baseline	–	0.826	–	0.854	–	0.827	–	83.20	–	0.929
	Suicide + bipolar	0.846	0.858	0.786	0.808	0.802	0.824	83.20	84.80	0.901	0.901
	Bipolar baseline	–	0.814	–	0.838	–	0.817	–	82.40	–	0.908
	Suicide + depress	0.784	0.784	0.775	0.775	0.779	0.779	80.00	80.00	0.881	0.881
	Depress baseline	–	0.797	–	0.820	–	0.801	–	80.80	–	0.893
	Suicide + ocd	0.760	0.776	0.742	0.764	0.748	0.769	77.60	79.20	0.867	0.866
	Ocd baseline	–	0.765	–	0.768	–	0.766	–	78.40	–	0.861
	Suicide + ptsd	0.851	0.851	0.854	0.854	**0.853**	**0.853**	86.40	86.40	0.942	0.942
	Ptsd baseline	–	0.841	–	0.858	–	0.847	–	85.60	–	0.938
	Suicide + sch	0.833	0.842	0.842	0.848	0.837	0.845	84.80	85.60	0.910	0.911
	Schizophrenia baseline	–	0.823	–	0.849	–	0.826	–	83.20	–	0.937
Expert	Suicide + ptsd	0.851	0.851	0.839	0.839	**0.845**	**0.845**	86.12	86.12	0.924	0.922
	Suicide baseline (expert)	0.639	–	0.646	–	0.641	–	66.53	–	0.643	-
Urgent/not urgent using tweets	Suicide + more_ptsd	0.878	0.878	0.907	0.907	0.884	0.884	88.80	88.80	0.959	0.959
	Suicide + more_depress	0.814	0.814	0.841	0.841	0.812	0.812	81.60	81.60	0.925	0.925
	Suicide + ptsd_depress	0.866	0.866	0.881	0.881	0.872	0.872	88.00	88.00	0.920	0.920
	Suicide + more_ptsd + embed	0.866	0.866	0.886	0.886	0.873	0.873	88.00	88.00	0.924	0.925
	Suicide + more_ptsd + empath	0.883	0.891	0.899	0.910	**0.889**	**0.898**	89.60	90.40	0.946	0.945
	Baseline (more_ptsd + depress)	–	0.840	–	0.868	–	0.829	–	83.20	–	0.965

The bold values represent the highest F1 score for suicide ideation and mental disorder detection.

## 5. Discussion

### 5.1. Task: Flagged/not flagged

According to Table 7 and when comparing the suicide ideation detection baseline F1 score (F1s) with the highlighted best F1 scores (for the task of predicting users with suicide ideation), we could identify that the proposed multi-task learning with soft and hard parameter sharing has given significantly better results. Overall, the results obtained for each of the multi-task learning experiments (e.g., “suicide + adhd,” “suicide + anxiety,” “suicide + more_ptsd,” “suicide + more_depress") have produced better F1 scores compared to the suicide ideation detection baseline. Also, when comparing the AUCs scores, the proposed architecture has produced a low false positive rate and a higher true positive rate. The best performances concerning the F1 scores (F1s and F1m) are from the tasks suicide ideation detection and PTSD mental disorder detection (“suicide + ptsd"). The tasks have reported better F1 scores when predicting users diagnosed with a single mental disorder (PTSD only) and multiple mental disorders (PTSD with one or more other mental disorders). Compared to the results obtained when users were diagnosed only with PTSD, users diagnosed with multiple mental disorders in addition to the primary diagnosis (PTSD) have produced better results. A similar pattern can be identified using the CLPsych 2015 Twitter data representing users with mental disorders, PTSD and depression. In general, using mental illness data has significantly improved predicting users susceptible to suicide ideation. However, the features being shared between users diagnosed with depression and users having suicidal thoughts are fewer in comparison to when using more users diagnosed with PTSD.

To identify the impact auxiliary inputs have on the prediction outcome, we used several EMAPTH categories and identified that the model performances could be further enhanced when using data from the same platform and different platforms. We obtained performance enhancements ranging from 0.5% to 1.0% with limited experiments using these auxiliary inputs. We also used pre-trained fastText embeddings to identify that the trained model could not generalize well on the unseen data compared to when using the randomly initialized word embeddings. Even though the performances could not surpass the highest F1 score, the model using the pre-trained word embeddings generalized well on unseen data to achieve AUC scores above 0.90 (for AUCs and AUCm).

**Figure 4:**
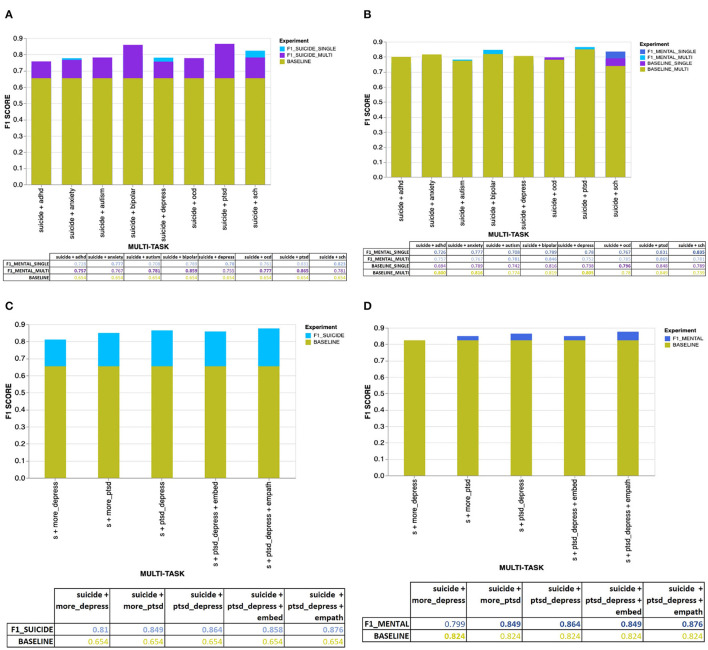
**(A)** Overall results for the suicide ideation detection task with users diagnosed with single or multiple mental disorders. **(B)** Overall results for the mental illness detection task with users diagnosed with single or multiple mental disorders. **(C)** Overall results for the suicide ideation detection task with users diagnosed with mental disorders using CLPsych 2015 data (either PTSD or depression). **(D)** Overall results for the mental illness detection task with data from users diagnosed with either PTSD or depression (using the CLPsych 2015 data).

**Figure 5:**
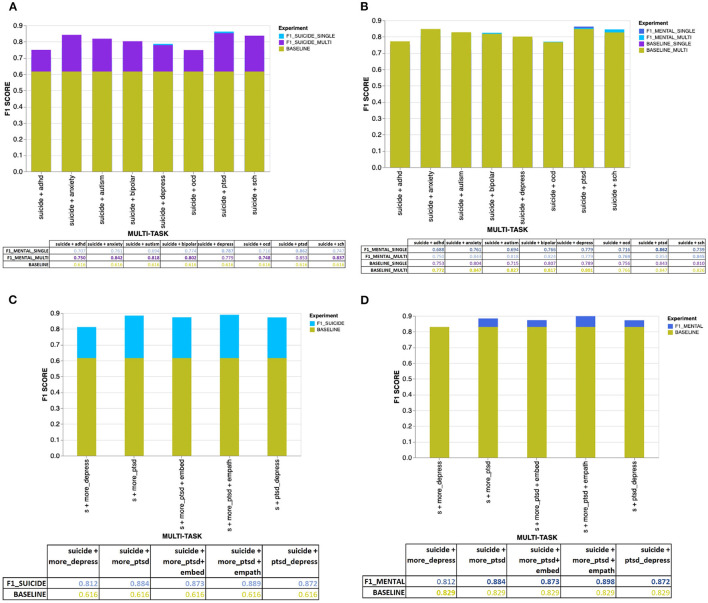
**(A)** Overall results for the suicide ideation detection task (for users requiring urgent attention) with users diagnosed with single or multiple mental disorders. **(B)** Overall results for the mental illness detection task with users diagnosed with single or multiple mental disorders. **(C)** Overall results for suicide ideation detection with data from users diagnosed with PTSD or depression (using the CLPsych 2015 data). **(D)** Overall results for the mental illness detection task with data from users diagnosed with either PTSD or depression (using the CLPsych 2015 data).

The Figures 4A–D, 5A–D provide an overview of the results obtained using the proposed MTL architecture using both same and cross-platform data. The F1 scores are from the tasks suicide ideation and mental illness detection, considering the results with (from multi-platform experiments) and without (from single-platform experiments) the auxiliary inputs. The outcome of each experiment is represented in the layered bar chart, where the labels mentioned in the chart legend indicate the F1 score for the related experiments.

*Experiments using data from a single social media platform:*

- F1_SUICIDE_SINGLE: F1 score for suicide ideation detection task given the users with a single mental disorder.
- F1_SUICIDE_MULTI: F1 score for suicide ideation detection task given the users with multiple mental disorders.
- BASELINE: Baseline F1 score for suicide ideation detection.
- F1_MENTAL_SINGLE: F1 score for mental illness detection task given the users with a single mental disorder.
- F1_MENTAL_MULTI: F1 score for mental illness detection task given the users with multiple mental disorders.
- BASELINE_SINGLE: Baseline F1 score for mental illness detection given the users with a single mental disorder.
- BASELINE_MULTI: Baseline F1 score for mental illness detection given the users with multiple mental disorders.

*Experiments using data from multiple social media platforms:*

- F1_SUICIDE: F1 score for suicide ideation detection task (for flagged/not flagged or urgent/not urgent).
- BASELINE: Baseline F1 score for suicide ideation / mental illness detection (for flagged/not flagged or urgent/not urgent).
- F1_MENTAL: F1 score for mental illness detection task (for flagged/not flagged or urgent/not urgent).

According to Figure 4A, we could see that users diagnosed with multiple mental disorders (apart from the user's primary diagnosis being anxiety, depression and schizophrenia) share more hidden features with users having suicide ideation than users diagnosed with a single mental disorder. In general, it is clear that, given the particular dataset, mental disorders have positively impacted the suicide ideation detection task in the MTL environment with soft and hard parameter sharing. Also, considering the eight mental disorders, different mental disorders have imposed a distinctive impact on suicide ideation detection. For example, users diagnosed with either PTSD, bipolar or schizophrenia tend to have shared more hidden features with users identified with suicidal thoughts than users with other mental disorders. All three mental disorders mentioned before have produced F1 scores >0.80 (for both F1s and F1m), with AUC scores between 0.88 and 0.97.

When computing the baseline for suicide ideation detection, we did not use a majority class baseline and instead used the same underlying multi-task learning architecture but for single task learning. When comparing our proposed baseline with the majority class baseline accuracy, it could be identified that, apart from a few occurrences, the proposed baseline model has produced better accuracies, especially for the mental disorder detection task. The majority class baseline accuracies for mental illness and suicide ideation detection are 74.4% (for flagged/not flagged) and 64% (urgent/not urgent). Apart from when detecting ADHD (for a single mental disorder) within the flagged/not flagged task, which has generated a slightly lower baseline accuracy (73.6%), the rest of the mental disorders have generated a baseline accuracy considerably higher than the majority class baseline (for single and multiple mental illness detection within both flagged/not flagged and urgent/not urgent tasks). However, the proposed baseline accuracies for suicide ideation detection are slightly less than the majority class baseline.

According to Figure 4B, when measuring the impact suicide ideation detection has on mental illness detection, we could identify that using data associated with certain mental disorders has not improved the model performances over the baseline predictions. For example, the tasks; “suicide + adhd,” “suicide + anxiety,” “suicide + depress,” and “suicide + ocd” have not managed to improve the F1 scores (F1m) for mental illness detection. Given the computed F1 scores and the baseline, it could be derived that the features shared between the two tasks have managed to improve only the performances of suicide ideation detection and have hindered the performances of mental illness detection. Even though the tasks were aligned, the information being transferred from one task to another has negatively impacted its performance (Wu et al., 2020). Hypothetically, users with certain mental disorders could have features shared with users with suicidal thoughts. However, on the contrary, users with suicidal thoughts might not have features that could be used to further distinguish users diagnosed with certain mental disorders from neurotypicals. The argument can be further extended to state that even though mental disorders such as depression strongly correlate with suicide ideation (Brådvik, 2018), given the SMHD dataset, the users diagnosed with depression might not have published content containing characteristics that link suicide risk with depression. For example, similar to the suicidality predictors identified among those who experienced depression, such as “depression history and severity,” “comorbid mental illness,” “help seeking,” and “socio demographic characteristics” (Handley et al., 2018), the neural network needs to extract certain distinctive features to discover the level of interrelatedness between suicide ideation and depression. Nevertheless, when using cross-platform data, it is clear that suicide ideation detection tasks had a noticeable influence on mental illness detection, where using data from users diagnosed with either PTSD or depression improved the mental illness detection F1 score from a strong baseline of 0.824 to 0.864 (s + ptsd_depress). Apart from the aforementioned mental disorders that have not gained any advantage concerning their detection given the multi-task learning environment, the remaining mental disorders, autism, bipolar, PTSD and schizophrenia, have reported improvement over the single-task baseline. Users diagnosed with multiple mental disorders, in addition to their primary diagnosis, autism, bipolar, and PTSD, have reported improved F1 scores.

When further evaluating Figures 4A, B, we could see that users diagnosed with multiple mental disorders have shared more features with users having suicidal thoughts than those diagnosed with a single mental disorder. Even when predicting the baseline for mental disorders, we could clearly distinguish that users diagnosed with multiple mental disorders in addition to their primary diagnosis have produced better F1 scores than those diagnosed with a single mental disorder. However, when it comes to users diagnosed with schizophrenia, the best F1 scores for suicide ideation and mental illness detection were discovered through content published by users with a single mental disorder rather than multiple mental disorders. Even though we could not describe the relationship between comorbidities and schizophrenia, it could be derived that specific psychosis characteristics unique to schizophrenia could have given the dataset its unique properties.

We used the expert annotated data from the UMD dataset during inference to further demonstrate how well the trained model generalizes on unseen data. Due to the limited number of instances annotated by the experts, the expert annotated data was used only for testing purposes rather than during training or the validation phases. We did not use the test data on all the models trained using eight mental disorders except with the best-performing model. According to Table 7 and section “expert,” we obtained 0.847 as the F1 score for both tasks. According to the AUC scores, we could identify how well the model has generalized on unseen data, even with a different class distribution than the crowdsourced test dataset.

### 5.2. Task: Urgent/not urgent

We conducted a similar set of experiments as previously mentioned in the task flagged/not flagged for the task urgent/not urgent. According to Table 8, sections “urgent/not urgent + single mental disorders,” “urgent/ not urgent multiple mental disorders,” and “urgent/not urgent using tweets,” we could identify that using the proposed multi-task learning model has produced significantly better results for the suicide ideation detection task for users that requires urgent attention. Similar to the flagged/not flagged task findings (when using the SMHD dataset), we did not find a significant improvement in mental illness detection (either single or multiple) compared to their respective baseline scores. Considering the reported metrics, we could identify that much of the knowledge being transferred is from the mental illness detection task to the suicide ideation detection, confirming the impact mental disorders have on users with suicidal thoughts who requires urgent attention. Overall, when predicting users with suicide ideation who require urgent attention, the features contributed by those diagnosed with PTSD have significantly improved the models' predictability. Even though EMPATH categories and fastText embeddings did not improve the prediction outcome when using data from the same social media platform, around 1% improvement was achieved using EMPATH categories from the CLPsych 2015 data. To further demonstrate how well the trained model (using data from the same platform) using our proposed architecture generalizes on unseen data, we used the UMD expert annotated data to predict users with suicide ideation and PTSD mental disorder (PTSD only). Table 8, section “expert” shows that both suicide ideation and mental illness detection tasks have achieved an F1 score of 0.845.

Figure 5 demonstrates the overall performances generated by the proposed MTL model on predicting users with suicide ideation that requires urgent attention in both single (Figures 5A, B) and multi-platforms (Figures 5C, D). The layered bar charts demonstrate the F1 scores obtained for suicide ideation and mental illness detection using data from users diagnosed with single or multiple mental disorders. In addition, individual baseline results for predicting users with suicide ideation or mental disorders are also included for comparison. According to Figures 5A, B, users diagnosed with PTSD have shared more features, with users having suicide ideation requiring urgent attention. Similar behavior can be identified (refer to Figures 5C, D) when using data from multiple platforms, where models trained with more data from users diagnosed with PTSD have produced better results than those trained with more users diagnosed with depression. The findings further highlight the lesser impact of the shared feature space between users with suicide ideation and depression. Following the task flagged/not flagged, using EMPATH categories has improved the best results when using CLPsych 2015 data, contrary to results obtained using data from the same platform. Apart from single mental disorders, users diagnosed with multiple mental disorders have shared more hidden features, with users having suicide ideation who requires urgent attention demonstrating the positive impact comorbidity of disorders has on suicide ideation. Even though all the mental disorders have improved the performances of suicide ideation detection, different mental disorders have demonstrated a varying degree of impact on detecting suicide ideation in a multi-task learning environment. Contrary to the flagged/not flagged task, users diagnosed with anxiety and autism, in addition to being diagnosed with multiple mental disorders, have contributed more toward identifying users with suicide ideation who require urgent attention.

According to Table 8, sections “urgent/not urgent single mental disorder,” “urgent/not urgent multiple mental disorders,” and Figure 5B, we could identify that, even though the task of mental illness has significantly improved suicide ideation detection, the detection of mental disorders has not vastly improved when trained alongside the suicide ideation detection task. For example, given the users diagnosed with either a single disorder or multiple mental disorders in addition to their primary diagnosis, prediction results of the disorders, ADHD, anxiety, autism and depression, did not improve over their respective baseline predictions. However, all the mental disorder prediction task accuracies have improved compared to the majority class baseline accuracy (0.64). Given the baseline predictions, we can derive that the knowledge shared between the two tasks has benefitted suicide ideation detection more than mental illness detection. Apart from the above-mentioned mental disorders, bipolar, OCD, PTSD and schizophrenia mental disorders have shared more knowledge with suicide ideation detection.

### 5.3. Comparison to related work

The results mentioned in Table 9 are compared based on the macro F1 score and have included the teams that have produced the top results for both flagged/not flagged and urgent/not urgent tasks in the CLPsych 2019 shared task. Due to the reason that the team “CAMH” has not provided a technical paper explaining their proposed solution, we could not extract further details on how they have achieved the best results for the task flagged/not flagged. We have highlighted our results, “MTL with soft and hard parameter sharing,” using data from single (Reddit) and multiple (Reddit and Twitter) social media platforms and emphasized the best results compared to the task participants. In comparison, our proposed architecture has generated F1 scores of 0.875 and 0.862 using data from the same platform and 0.876 and 0.889 from two different platforms for flagged/not flagged and urgent/not urgent tasks. The overall results obtained using the proposed architecture ranked second in the task flagged/not flagged and first in urgent/not urgent, with an overall performance gain of 5% over the reported best results. Further analysis shows that our proposed architecture has produced consistent results over both tasks.

**Table 9:** Related work comparison for suicide ideation detection (using SMHD and CLPsych 2015 Twitter data).

**Submissions**	**F1(flagged/not flagged)**	**F1(urgent/not urgent)**
Matero et al. (2019)	0.821	0.816
CAMH	**0.91**	0.812
Iserman et al. (2019)	0.848	0.775
Mohammadi et al. (2019)	0.843	0.718
MTL with soft and hard parameter sharing (using SMHD data)	0.875	**0.862**
MTL with soft and hard parameter sharing (using Twitter data)	0.876	**0.889**

The bold values represent the highest F1 score for suicide ideation and mental disorder detection.

To evaluate how well the proposed solution has predicted users with mental disorders, we compared our results with Cohan et al. (2018), who created the SMHD dataset. Because the authors have used the complete dataset and based on the assumption that the reported F1 score is computed on the positive class, we could not directly compare our results with the authors' results. However, using a randomly selected sample from the SMHD dataset for train, validation and test, we achieved a macro F1 score of 0.875 for predicting users with PTSD. Even though the mental illness detection results using the CLPsych 2015 dataset could not be compared directly with the CLPsych 2015 shared task results due to an inadequate number of instances and architectural requirements, we identified competitive outcomes on multiple randomly sampled test datasets predicting users with PTSD or depression. For the tasks flagged/not flagged and urgent/not urgent, our proposed model has generated AUC scores of 0.959 and 0.945, respectively.

### 5.4. Applications

Given the sensitive nature of the data and especially the ethical and privacy concerns, it is crucial to understand the factors that must be considered when introducing specific solutions into clinical environments. For example, detecting an individual with suicide risk is insufficient unless linked with an intervention mechanism (Linthicum et al., 2019). Over the years, only a few applications were implemented successfully to predict users with suicide ideation and mental disorders as an initial part of the intervention mechanism. Two applications that failed to protect users' privacy were Samaritans Radar (Samaritans, 2015) and Koko (Jaroszewski et al., 2019). However, Milne et al. (2019) introduced a machine learning classifier to prioritize forum posts based on their severity and managed to reduce the response delays. Similar to Milne et al. (2019), we could introduce our proposed model to a platform where consenting users can visit for mental health support and will get prioritized based on the level of risk associated with their posted content. However, these platforms must be regularized by trained moderators who handle users with less critical concerns, while qualified mental health professionals will handle severe incidents. In addition to these support forums, we will investigate the avenues of using our proposed architecture in other platforms (e.g., subreddits unrelated to mental health) under strict ethical and privacy guidelines.

### 5.5. Conclusion

This research highlights the importance of early detecting users with mental disorders and suicide ideation. We proposed a multi-task learning architecture to identify at-risk users and produced state-of-the-art results in predicting users with suicide ideation. Mental disorders and suicide are worldwide health problems where lack of support for people in need has deteriorated their mental and physical wellbeing. Given the popularity and information richness of social media platforms, it was identified that such platforms could be used to initiate mental health care if ethical principles are respected. Using our proposed multi-task learning architecture, we proved that users with suicide ideation or mental disorders could be identified with low false positive and false negative rates.

We decided to use multi-task learning to early detect social media users with suicidal thoughts or mental disorders with inspiration from studies conducted by public health researchers to identify the relationship between suicide risk and mental disorders. The research was performed in two phases, one where suicide ideation and mental illness data were obtained from the same platform and the second from multiple platforms. The experiments were further categorized into tasks flagged/no flagged and urgent/not urgent based on the severity level determined by the posted content. Within each category, experiments were conducted to identify the correlation between suicide ideation and mental disorders, which could be either single or multiple (comorbidity), using data from the same social media platform. Finally, we used data from two different social media platforms with different distributions to identify the extent to which the knowledge can be shared between the tasks of suicide ideation and mental illness detection. With research conducted using different multi-task learning architectures such as hard parameter sharing and soft parameter sharing, we identified that a combination of both hard and soft parameter sharing is more effective in discovering hidden shared features between users who self-declared mental disorders and users having suicidal thoughts. The best-performing models were further tested to discover the impact predetermined auxiliary inputs have on suicide ideation and mental illness detection. With limited experiments, it was discovered that the overall performance could be improved, mainly when predicting users with suicide ideation.

Extensive experiments identified a strong correlation between suicide ideation and certain mental disorders. Among the eight tested disorders, users whom self-declared PTSD, bipolar or schizophrenia as the primary diagnosis shared more features with users having suicidal thoughts. A similar outcome was recognized when predicting users with suicide ideation who require urgent attention, where users whom self-declared only PTSD shared more features with users having suicidal thoughts. The impact of comorbidity on suicide ideation detection was identified throughout all the experiments. Primary diagnosis combined with one or more disorders has resulted in better predictions than when using data from users diagnosed with a single mental disorder. The models trained using the UMD and SMHD datasets were further tested using the UMD expert annotated dataset to determine how well the trained models have generalized. The tests identified that the trained models have generalized well when predicting users with suicide ideation and mental disorders with an F1 score > 0.84.

During the second phase of our research, we could not conduct experiments using content from users diagnosed with a single mental disorder. Instead, we randomly sampled data from users diagnosed with PTSD and depression. Similar to phase one experiments, we identified that users with suicide ideation do share features with users diagnosed with either PTSD or depression. When predicting users with suicide ideation, we identified that increasing the number of users diagnosed with depression in the sample reduced the model performance significantly (about 8% in the F1 score). Similar to when using data from the same platform, it could be identified that a sample of users with different mental disorders share more features with suicide ideation than having more data from users diagnosed with a single mental disorder. However, when identifying users with suicide ideation who require urgent attention, users diagnosed with PTSD shared more features with at-risk users than when sampling more users who self-declared depression. Using several selected EMPATH categories as auxiliary inputs also enhanced the prediction capabilities of the model.

### 5.6. Limitations and challenges

One of the critical limitations in our research is the lack of data when training the model. The need for more data is identified throughout training, where the trained models for mental illness and suicide ideation detection tend to overfit the training data. Having more data to train the suicide ideation detection task could have also improved the overall predictions during inference. However, we obtained a well-generalized model using limited instances and different strategies to overcome model overfitting.

In our experiments, we could not use state-of-the-art transformer-based architectures due to the inability of the models to process longer sequences. For our research, we concatenated all the posts published by each user. As a result, the average sequence length per user for all the datasets was above the maximum sequence length expected by most transformer-based architectures. Even though the posts are concatenated according to their timestamps, we could not identify the exact locations within the sequence indicating the signs of mental disorders or suicidal thoughts. Due to this reason, truncating the longer sequences to accommodate the model requirements could significantly increase the number of false positives and negatives. Even though specific models are being introduced to overcome the sequence length limitations, they are either insufficient to accommodate the required sequence length for our experiments or, even if it does manage to accommodate a longer sequence, it requires more computational resources to train the model. Even though we did not use transformer-based architectures, we obtained better results than research using architectures such as BERT (e.g., Matero et al. 2019).

### 5.7. Future work

The research can be further extended by including users with mental disorders not incorporated in the used datasets to identify their relationship with suicide ideation. Apart from the hyperparameters we have fine-tuned to obtain optimal model performance, we can explore many other hyperparameters that can be optimized to enhance overall performance. Also, the potential for more research exists using current state-of-the-art transformer-based architectures by exploring the methods to overcome sequence length limitations.

Because our research did not focus on predicting the risk categories of users (“no,” “low,” “moderate,” and “severe") due to the research objective and architectural requirements, further research can be done to predict these risk categories by measuring the impact different mental disorders have on each category. We will further investigate the UMD dataset by structuring it in a way that can be used to analyze the temporal change in the user-generated data. As people's mental health conditions could change over time, it is vital to assess the mental state of an individual (in our case, using text data) over a certain period rather than focusing on a limited time frame.

## Data availability statement

The data analyzed in this study is subject to the following licenses/restrictions. The datasets are available for research. We obtained the ethics approval certificate for the CLPsych 2015 dataset and the University of Maryland Reddit Suicidality Dataset. We provided a signed data usage agreement to acquire the Self-Reported Mental Health Diagnoses dataset from Georgetown university. During our research, we followed strict ethical guidelines to ensure the anonymity and privacy of the data. Our research does not involve any intervention and has focused mainly on the applicability of machine learning models in determining users susceptible to mental disorders and suicide ideation using the before mentioned datasets. Also, we have not included examples from the datasets in any of our publications. Requests to access these datasets should be directed at: http://users.umiacs.umd.edu/~resnik/umd_reddit_suicidality_dataset.html, https://ir.cs.georgetown.edu/resources/smhd.html, and https://www.cs.jhu.edu/~mdredze/clpsych-2015-shared-task-evaluation/.

## Author contributions

PB designed and run the experiments and wrote the first draft of the paper. DI defined the project and helped with revising the paper. Both authors contributed to the article and approved the submitted version.

## Footnotes

^1^With extensive experiments, we identified 10 epochs as the optimal number of epochs for the model to be trained, considering the factors such as training loss, validation loss and variance.
